# Emphysematous Pyelonephritis Caused by Klebsiella pneumoniae Complicated With Multiple Metastatic Abscesses and Transient Respiratory Arrest: A Case Report

**DOI:** 10.7759/cureus.105828

**Published:** 2026-03-25

**Authors:** Takahiro Aoyagi, Shuhei Ishii, Yukihisa Owari, Gaku Takahashi, Wataru Obara

**Affiliations:** 1 Department of Urology, Iwate Prefectural Miyako Hospital, Miyako, JPN; 2 Department of Urology, Iwate Medical University, Shiwa, JPN; 3 Department of Emergency Medicine, Iwate Medical University, Shiwa, JPN

**Keywords:** blood purification therapy, emphysematous pyelonephritis (epn), klebsiella pneumoniae (kp), multiple lung abscesses, prostatic abscess

## Abstract

Emphysematous pyelonephritis (EPN) is a life-threatening urinary tract infection that often requires prompt source control, including drainage, in addition to intensive supportive care. We report a severe case in which immediate invasive source control measures were not feasible; however, the patient survived with intensive multidisciplinary management. A 50-year-old man with poorly controlled diabetes mellitus presented with back pain and was diagnosed with left-sided EPN based on computed tomography. Owing to his overall critical condition, invasive renal or prostatic drainage was not performed. Blood and urine cultures revealed *Klebsiella pneumoniae*. During the clinical course, he developed multiple lung abscesses. On day 11, the patient experienced transient respiratory arrest, requiring mechanical ventilation and chest drainage. The patient gradually improved without nephrectomy or urinary diversion and was discharged on day 65 with functional recovery. Even when early invasive source control measures are not feasible in severe EPN, appropriate intensive multidisciplinary management may lead to favorable outcomes.

## Introduction

Emphysematous pyelonephritis (EPN) is a severe urinary tract infection characterized by gas formation within the renal parenchyma and surrounding tissues [1]. It occurs most frequently in patients with diabetes mellitus and may progress to severe sepsis and multiorgan failure [1,2]. The most common causative organisms are *Escherichia coli* and *Klebsiella pneumoniae (K. pneumoniae)*.

The standard management of EPN includes prompt administration of broad-spectrum antimicrobial therapy and, in severe cases, invasive source control such as percutaneous drainage or nephrectomy [2,3]. However, some patients present with hemodynamic instability or multiorgan failure, making invasive procedures unsafe or impractical.

In the present case, EPN caused by *K. pneumoniae* was complicated by prostatic and multiple metastatic lung abscesses, ultimately leading to transient respiratory arrest. Despite the inability to perform immediate invasive drainage, the patient recovered after intensive multidisciplinary management.

EPN complicated by metastatic abscesses and respiratory arrest is rare and represents a particularly severe clinical presentation. In addition, hypervirulent *Klebsiella pneumoniae* (hvKp) has been increasingly recognized as a cause of invasive infections with metastatic spread. Therefore, this case highlights both the severity and clinical significance of EPN associated with possible hvKp infection.

## Case presentation

**Figure 1:**
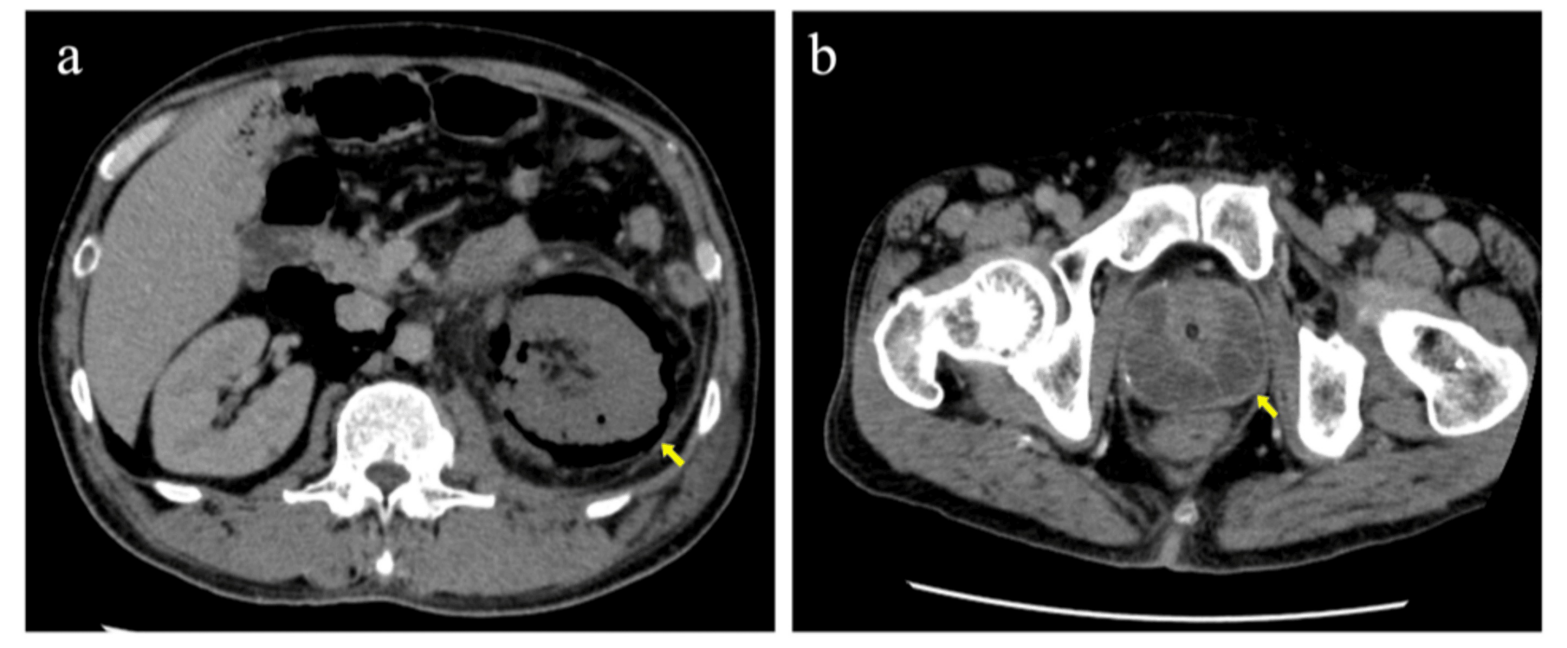
Contrast-enhanced computed tomography findings on admission. (a) Contrast-enhanced axial computed tomography on admission demonstrating extensive gas formation in the left renal parenchyma and perirenal space (yellow arrows). (b) Contrast-enhanced axial computed tomography demonstrating multiple low-density lesions with internal septations in the prostate gland (yellow arrow), consistent with a prostatic abscess.

A 50-year-old man with a history of poorly controlled diabetes mellitus presented to a local hospital with back pain. He also reported general fatigue and decreased appetite before admission, although no obvious urinary symptoms were noted. Plain computed tomography (CT) revealed extensive gas formation in the left renal pelvis, renal parenchyma, and perirenal space, extending into the retroperitoneum. His initial vital signs were as follows: heart rate of 114 beats per minute, blood pressure of 86/54 mmHg, respiratory rate of 18 breaths per minute, temperature of 36.8°C, and SpO₂ of 98% on room air. Physical examination revealed left costovertebral angle tenderness without signs of peritoneal irritation. Laboratory testing showed acute kidney injury according to the Kidney Disease: Improving Global Outcomes (KDIGO) criteria and an HbA1c of 12.8%. The patient was transferred to our hospital with a diagnosis of EPN associated with poorly controlled diabetes mellitus. On arrival, his vital signs were as follows: heart rate of 106 beats per minute, blood pressure of 113/81 mmHg, respiratory rate of 17 breaths per minute, temperature of 36.9°C, and SpO₂ of 97% on 2 L/minute oxygen. Laboratory findings were as follows: white blood cell count was 11,600/μL, platelet count was 99,000/μL, and C-reactive protein (CRP) was 28.22 mg/dL. The serum creatinine was 2.62 mg/dL, consistent with acute kidney injury. Serum sodium level was 118 mEq/L, and serum potassium level was 4.5 mEq/L. Arterial blood gas analysis revealed a pH of 7.47, partial pressure of carbon dioxide of 31 mmHg, partial pressure of oxygen of 127 mmHg, bicarbonate level of 22 mEq/L, and a serum lactate of 1.7 mmol/L. The PaO₂/FiO₂ ratio was 530.8. The initial Sequential Organ Failure Assessment score was 9. Contrast-enhanced CT demonstrated extensive gas in the left kidney and perirenal space extending to the contralateral perirenal region (Figure 1a). Multiple low-density lesions were observed in the prostate gland (Figure 1b). No urinary tract obstruction was observed. The primary diagnosis was EPN complicated by severe sepsis and transient hypotension.

**Figure 2:**
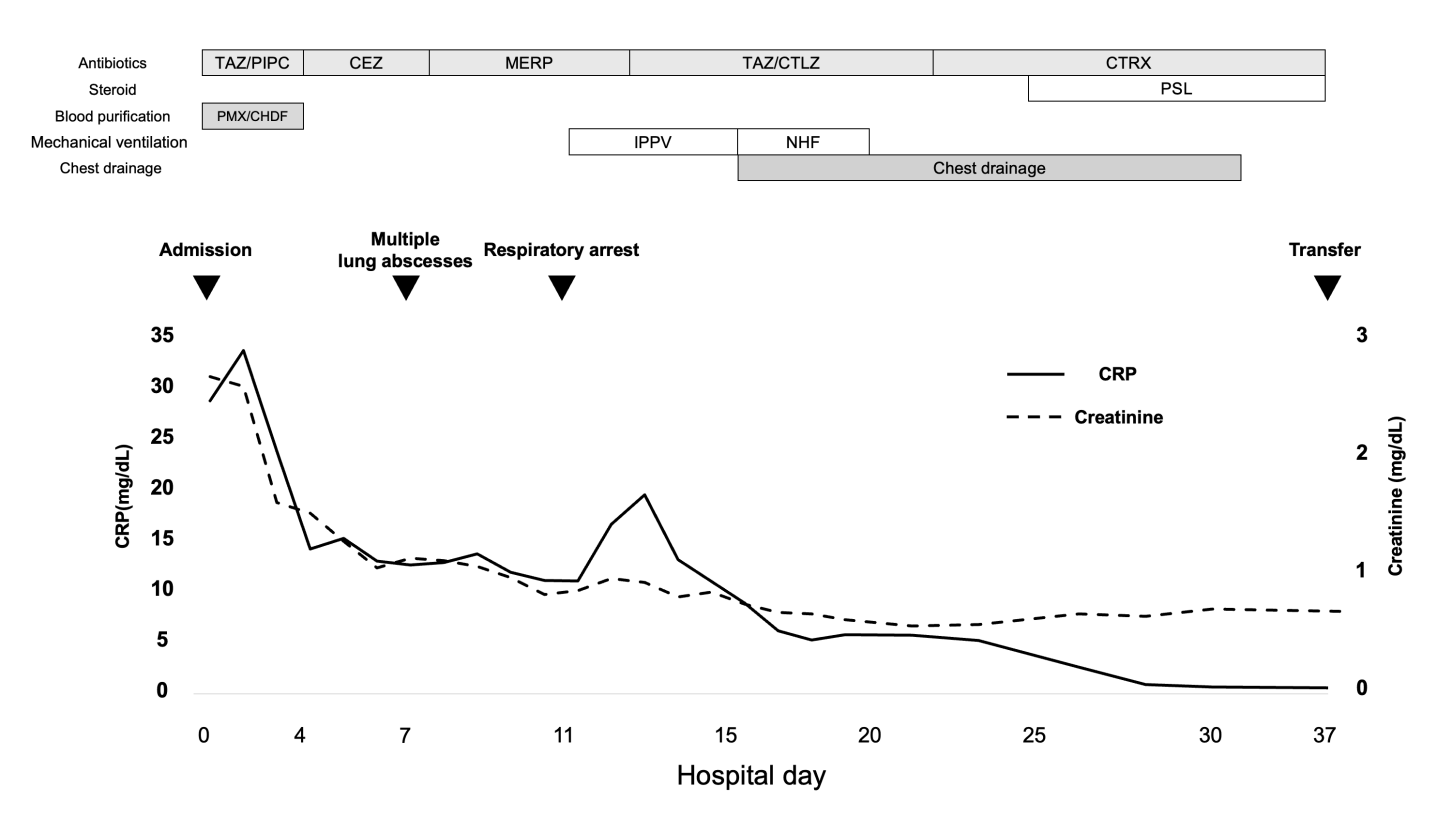
Clinical course and treatment timeline during hospitalization. Temporal changes in C-reactive protein (CRP) and serum creatinine during hospitalization are shown. CRP levels decreased following antimicrobial therapy, while serum creatinine gradually improved with supportive care including continuous renal replacement therapy. The timeline also illustrates the relationship between clinical events and therapeutic interventions, including antimicrobial therapy adjustments, blood purification therapy (continuous renal replacement therapy and polymyxin B hemoperfusion), respiratory management including mechanical ventilation, and chest drainage for multiple lung abscesses.

The clinical course is shown in Figure 2. Owing to severe sepsis and progressive organ dysfunction, invasive procedures were not feasible, and prostatic drainage was not performed. Additionally, percutaneous drainage of the perirenal lesion was not indicated because imaging demonstrated predominantly emphysematous changes without a localized fluid collection amenable to drainage. Therefore, empiric antimicrobial therapy with tazobactam/piperacillin was initiated. Continuous renal replacement therapy and polymyxin B hemoperfusion were initiated as part of intensive supportive care.

**Figure 3:**
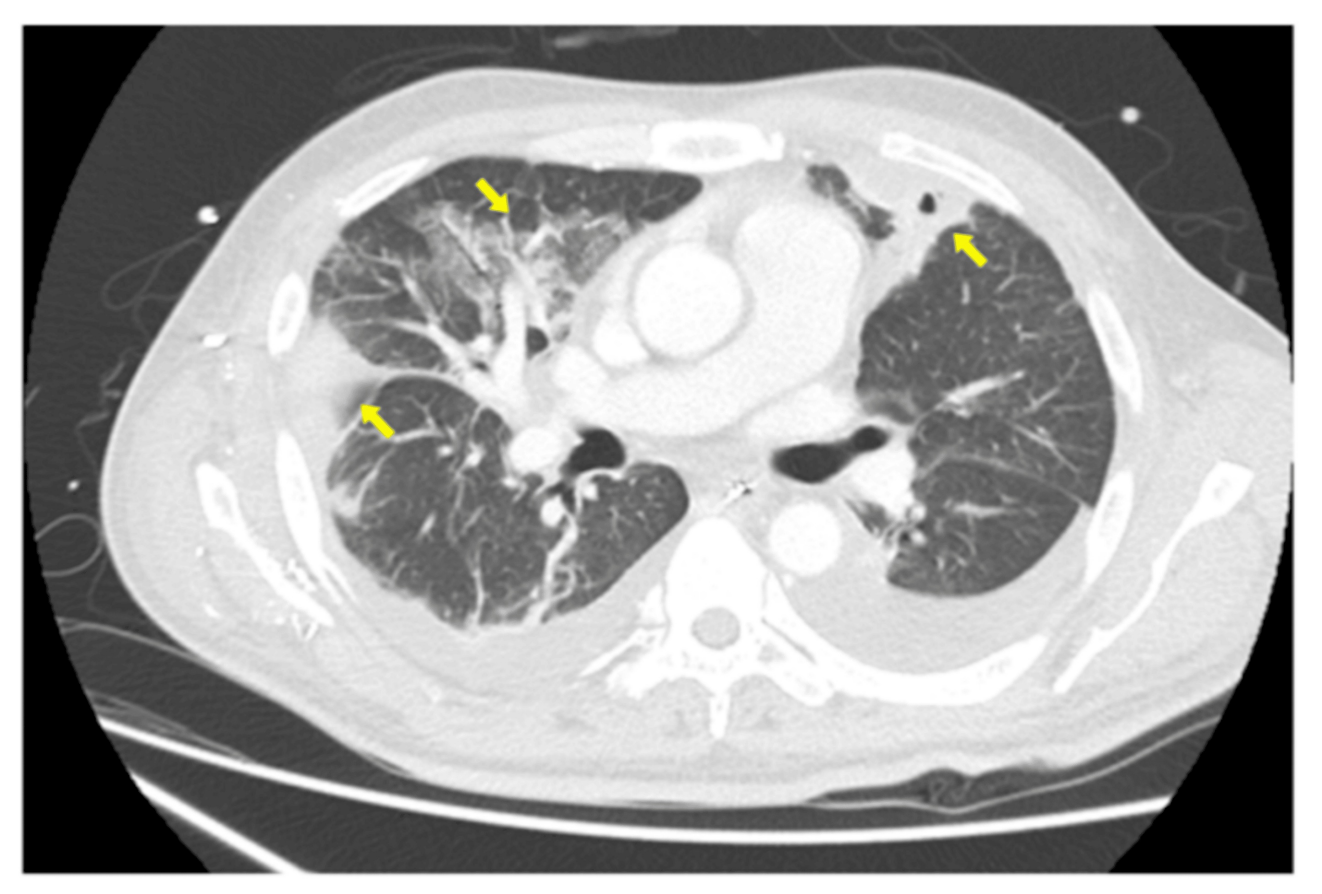
Follow-up contrast-enhanced axial computed tomography of the chest. This image demonstrates multiple lung abscesses on hospital day seven.

Blood and urine cultures revealed *K. pneumoniae*, of which the string test results were negative. Antimicrobial therapy was switched to cefazoline. Follow-up CT on hospital day seven revealed multiple lung abscesses (Figure 3). On day 11, the patient experienced a transient respiratory arrest and required tracheal intubation, which led to mechanical ventilation. On day 15, chest drainage was performed for the lung abscesses, and his general status improved. The patient was successfully extubated and gradually recovered. Follow-up CT demonstrated resolution of gas formation and spontaneous regression of the prostatic abscess. Nephrectomy, ureteral stenting, or percutaneous nephrostomy was not performed. He was transferred back to the referring hospital for rehabilitation on day 37 and was discharged on day 65.

During outpatient follow-up, the patient showed no recurrence of infection or abscess formation. Follow-up CT revealed complete atrophy of the left kidney with suspected dense adhesion to the iliopsoas muscle. The prostatic abscess resolved without invasive intervention. Because the infection was adequately controlled, and the patient remained clinically stable, additional surgical intervention was not performed. The patient successfully returned to normal activities of daily living.

## Discussion

EPN is a severe urinary tract infection characterized by gas formation within the renal parenchyma and surrounding tissues, and most commonly occurs in patients with diabetes mellitus [1]. Although nephrectomy was previously considered the standard treatment for severe cases, recent studies have suggested that conservative management with appropriate antimicrobial therapy and intensive supportive care can preserve renal function and improve survival [2-4]. The temporal changes in CRP and serum creatinine in the present case reflected the patient’s response to treatment. The marked elevation of CRP indicated severe systemic inflammation, which gradually improved following antimicrobial therapy. Similarly, renal function improved with intensive supportive care, including continuous renal replacement therapy.

*K. pneumoniae* is one of the major causative organisms of EPN. hvKp is a distinct clinical entity capable of causing invasive infections with metastatic abscess formation [5,6]. Although microbiological confirmation of hvKp was not obtained in this case, the presence of multiple metastatic abscesses suggests an invasive infection consistent with hvKp-related disease. Previous reports have described EPN caused by hvKp, which is sometimes accompanied by metastatic infections requiring invasive management [7]. Although hvKp infections can occur in healthy individuals, diabetes mellitus has been identified as an important risk factor for severe disease and metastatic spread [5]. Prostatic abscesses caused by *K. pneumoniae* have also been reported in the literature [8]. Although the string test was negative and a genetic test was not performed, hvKp infection was suspected because the patient developed multiple metastatic abscesses. Although a positive string test result is not required to diagnose hvKp [9], the lack of a microbiological confirmation of hvKp is a limitation of this study.

In patients with severe EPN, early source control measures are generally recommended [2,3]. At the time of admission, the patient was hemodynamically unstable, and invasive drainage procedures were considered unsafe. Additionally, imaging did not demonstrate a localized fluid collection amenable to drainage. Despite the inability to perform immediate surgical source control, intensive supportive care, including blood purification therapy and respiratory management, resulted in clinical improvement. Although delayed source control measures may have contributed to disease progression, the patient ultimately recovered without nephrectomy or urinary diversion. hvKp is associated with invasive infections and metastatic abscess formation. The typical metastatic complications include meningitis, endophthalmitis, and necrotizing fasciitis [10]. However, disseminated infections with multiple lung abscesses caused by hvKp have also been reported and can lead to fatal outcomes [6,10]. This case suggests that, even when immediate invasive intervention is not feasible, aggressive multidisciplinary intensive care may still result in favorable outcomes.

## Conclusions

Immediate invasive source control may not be feasible in severe infections such as EPN. This case suggests that intensive supportive care may achieve favorable outcomes even when early invasive intervention is not feasible.
